# No harm in being self-corrective: Self-criticism and reform intentions increase researchers’ epistemic trustworthiness and credibility in the eyes of the public

**DOI:** 10.1177/09636625211022181

**Published:** 2021-06-20

**Authors:** Marlene Sophie Altenmüller, Stephan Nuding, Mario Gollwitzer

**Affiliations:** Ludwig-Maximilians-Universität München, Germany

**Keywords:** credibility, open science, reforms, self-criticism, trust

## Abstract

Science should be self-correcting. However, researchers often hesitate to admit errors and to adopt reforms in their own work. In two studies (overall *N* = 702), we test whether scientific self-criticism and reform intentions expressed by researchers damage or rather improve their reputation in the eyes of the public (i.e. perceivers). Across both studies, such self-correction (compared to no self-correction) increases perceivers’ epistemic trustworthiness ascriptions, credibility perceptions, and willingness to further engage with science. Study 2 revealed that these effects were largely driven by the no self-criticism condition. In addition, researchers’ commitment to implementing reforms had positive effects and rejecting reforms had negative effects on perceptions, irrespective of the extent of these reforms. These findings suggest that researchers’ fear that self-criticism and expressing reform intentions may damage their reputation may be unfounded.

Humans fail and err all the time in their daily lives. Yet, admitting failures and errors is psychologically costly: doing so threatens one’s self-image and the desire to make a favorable impression upon others. While there are certainly contexts in which people’s hesitation to admit potential errors and to change their work routines is psychologically comprehensible, science is no such context: here, a self-corrective mind-set (i.e. admitting flaws and intending to improve one’s routines) is crucial. The basic idea of scientific progress is that by constantly correcting previous work and improving future work, researchers increase the likelihood of detecting the “truth” (or, at least, a robust phenomenon) and, thus, their own and other stakeholders’ confidence that their findings are trustworthy and credible.

However, empirical findings suggest that the ever-doubtful and self-correcting scientist is an ideal and that, when it comes to admitting flaws and failures, researchers behave just as “normal” people do: they hesitate to do so for the sake of protecting their self-image and their reputation (Bishop, 2018; Fetterman and Sassenberg, 2015; Rohrer et al., 2021; van der Bles et al., 2020). Thus, the question is whether these concerns are justified. How does the general public react to researchers who admit that their work may have been faulty and that they are willing to implement reforms aimed at improving the quality of their future work? Would the public place more versus less trust in researchers who admit such prior faults and/or who express reform intentions, and does the extent of these intended reforms play a role here? The two studies presented here were designed to find answers to these questions.

Trust in science can be conceptualized on different levels: trust in science as a whole and trust in individual researchers and their work. Judging a scientist to be a reliable source of knowledge is known as epistemic trust, which includes a cognitive aspect—expertise—and two affective aspects—benevolence and integrity (Fiske and Dupree, 2014; Hendriks et al., 2015; McAllister, 1995; Mayer et al., 1995; Neal et al., 2012). Expertise means that one is perceived as able and competent with regard to their (scientific) work; benevolence means that one is perceived as having the best in mind for others/society; and, finally, integrity means that one is perceived as adhering to prescriptive rules and principles. An additional element of trust in science, which is not directly reflected by any of the three interpersonal facets mentioned above, is credibility—people’s willingness to accept a scientific finding as true and integrating it in their own understanding of the world. Both epistemic trustworthiness and credibility are relevant for maintaining general trust in science on a societal level.

Previous findings suggest that self-criticism has positive as well as negative effects on people’s trust in scientists. For instance, Hendriks et al. (2016) found that expertise ascribed to a science blogger was lower when the blogger admitted (vs did not admit) an error in one of their blog entries, while perceived integrity as well as benevolence were higher. Notably, the error that the blogger admitted was not related to the research *per se* (i.e. the methodological quality of the study), but rather to how they communicated about it (i.e. overgeneralization of results in a science journalism piece). It is unclear, however, how admitting doubts about one’s own research might influence the public’s trust in scientists. In addition, it is unclear whether such self-criticism also affects credibility judgments. Here, effects are plausible in both ways: On one hand, admitting doubts about past work might imply incompetence and, hence, decrease expertise and credibility judgments. On the other hand, being self-critical and noticing and disclosing potential flaws in one’s previous work might indicate a more attentive approach to future research projects, which leads to more confidence that this work will produce sound scientific results. In addition, a self-critical approach to one’s research demonstrates commitment to a certain scientific attitude of constantly challenging and updating scientific knowledge, even at the cost of questioning oneself.

Regarding the effect of reform intentions on the public’s trust in science, previous studies yielded an inconsistent pattern: recent studies support the idea that successful replications increase laypeople’s trust in science (Hendriks et al., 2020; Wingen et al., 2020). However, learning about specific reforms can have null or even backfiring effects. For instance, Wingen et al. (2020) found that increasing transparency (e.g. by means of preregistrations, open data, and open materials) or providing explanations for the “replicability crisis” in psychology had no effect on laypeople’s trust in psychological science. Anvari and Lakens (2018) even found that participants expressed less trust in psychological science after learning about suggested reforms. The authors discuss three explanations for this backfiring effect: first, respondents may have been negatively surprised that the proposed reforms are not already common practice; second, their manipulation of reform implementation might have been problematic (i.e. their “reform” vignette began by talking about replication failures, while the other vignettes began by talking about the history of psychology, which might have elicited a stronger negative response in the reform condition); third, participants might have judged the reforms to be too weak. These inconclusive results call for more research on the effect of reform intentions on epistemic trustworthiness and credibility judgments.

Expanding our main focus on trustworthiness and credibility,^1^ we will also investigate the effect of self-criticism and reform intentions on participants’ willingness to engage with science. Disclosure of uncertainties and doubts has been found to not only have a mixed impact on perceptions of trustworthiness and credibility (e.g. Hendriks et al., 2016; Jensen, 2008; van der Bles et al., 2020) but also to increase the public’s interest in science and in new technologies (Retzbach and Maier, 2015). Thus, self-correction in science—expressing self-criticism and reform intentions—might also influence laypeople’s interest and make them want to engage more with science.

In this article, we report two preregistered studies investigating the effects of researchers’ self-criticism and reform intentions on their epistemic trustworthiness, the credibility of their future findings, and the public’s willingness to engage further with these researchers and their findings. For both studies, we report how we determined our sample size, all data exclusions (if any), all manipulations, and all measures (Simmons et al., 2012). All materials, the anonymized data, and analyses are available online (https://osf.io/yhsbp/).

## 1. Study 1

In Study 1, we compare the effect of self-criticism (yes vs no) and reform intentions in varying degrees: As previous studies regarding the perception of reforms focused on reforms in general (Anvari and Lakens, 2018) or on specific reform approaches (Wingen et al., 2020), we manipulate the extent of these reforms irrespective of their specific content (see “Methods” section; no, minor, or major reforms). This way, we account for two issues related to previous research in this area: First, we investigate the effect of the extent to which a researcher promises to implement reforms in their research on laypeople’s trust and credibility perceptions (Anvari and Lakens, 2018). Second, we try to get a more generalizable picture of the effects of reform intentions (as the findings will not be specific to distinct reforms, but rather get at the general willingness to implement reforms of differing degrees).

In Study 1, we also explore possible interaction effects of self-criticism and reform intentions on epistemic trust, credibility judgments, and willingness to engage. First, it is possible that self-criticism of previous work and reform intentions are perceived independently from each other, and, thus, yield two main effects on trust, credibility, and willingness to engage. Second, it is also possible that expressing doubts about one’s prior work makes reform intentions more reasonable and more credible, which would result in a synergetic interaction between self-criticism and reform intentions on trust, credibility, and willingness to engage. Third and finally, self-criticism of past work might lead to reform intentions for future work being perceived as a mandatory consequence; therefore, self-criticism followed by a refusal to implement future reforms may be perceived as inconsistent, and expressing reform intentions preceded by full-blown confidence in one’s prior results may be perceived as “cheap talk” and, thus, lead to particularly low levels of trust, credibility, and willingness to engage (i.e. an ordinal interaction between self-criticism and reform intentions).

### Methods

#### Experimental manipulation

We conducted an online study using a 2 (self-criticism: yes/no) × 3 (reform intentions: no/minor/major) full between-subject design. After obtaining informed consent, participants were asked to read an alleged online interview with Dr. Romberg,^2^ a psychological researcher, who talks about a past study he conducted. At one point during the interview, Dr. Romberg stated (without being asked) either that “. . . looking back on this study today, I admittedly doubt these findings. The results are probably not quite right, because according to my current methodological knowledge this study has some weaknesses” (self-criticism) or that “. . . looking back on this study today, I do not doubt these findings. The results are probably right, because according to my current methodological knowledge this study has no weaknesses” (no self-criticism).

Next, being asked explicitly about the on-going “open science” reform discussion in psychological science, Dr. Romberg describes some reforms in lay-terms (transparency through open data and preregistrations) and explains how they should work (enhanced reproducibility and constructive exchange, early detection of mistakes, higher reliability of findings). Then the interviewer asks Dr. Romberg how he judges these reforms in regard to his own research, to which he either states: “To be honest, I do not think these reforms are necessary for research on group processes. Therefore, I won’t apply any of these currently discussed reforms in my future research” (no reform intentions); “To be honest, I think these reforms are partly necessary for research on group processes. Therefore, I will apply some of these currently discussed reforms in my future research” (minor reform intentions); or “To be honest, I think these reforms are necessary for research on group processes. Therefore, I will apply many of these currently discussed reforms in my future research” (major reform intentions).

#### Dependent variables

After completing two attention check questions (“Which optimal group sizes did Dr. Romberg’s study show?”; “Which topic does Dr. Romberg want to investigate next?”), participants rated Dr. Romberg’s trustworthiness with the Muenster Epistemic Trustworthiness Inventory (METI; Hendriks et al., 2015), consisting of 14 opposite adjective pairs measuring expertise (e.g. competent–incompetent, Cronbach’s α = .94) and integrity and benevolence (e.g. honest–dishonest, Cronbach’s α = .95)^3^ on 6-point bipolar scales. Next, they rated the perceived credibility of Dr. Romberg’s future research on four items developed specifically for the purpose of this study based on theoretical assumptions (e.g. Anvari and Lakens, 2018), including cognitive as well as behavioral indicators of credibility (6-point Likert-type scale ranging from 1 = “not at all” to 6 = “absolutely”; for example, “I think, future research findings by Dr. Romberg will be credible,” “I will try to consider future research findings by Dr. Romberg in my daily life”; Cronbach’s α = .83). Finally, participants’ willingness to further engage with Dr. Romberg’s research (“I intend to register for the free account to be able to read the rest of the article”), support for public funding (“Dr. Romberg’s future research deserves public funding”), and likeability (“I like Dr. Romberg”) were assessed on 6-point Likert-type scales ranging from 1 = “not at all” to 6 = “absolutely.”

#### Other measures

In the final section, participants completed two manipulation comprehension check questions (“Did Dr. Romberg criticize his own previous study about group size?”; “Does Dr. Romberg want to apply reforms for his future research?”) by selecting either “yes” or “no.” If participants believed Dr. Romberg wanted to apply reforms, participants were asked about the assumed extent of these reforms (response options were “some reforms,” “many reforms,” or “don’t know”). To control for prior knowledge, we asked participants whether they had heard about the “replication debate” in psychology before, and, if yes, how much they knew about it on a 6-point Likert-type scale ranging from 1 = “not much” to 6 = “a lot.” Furthermore, we measured participants’ general public engagement with science (PES) using two scales that had been used in previous research (BBVA Foundation, 2011): a five-item scale measuring engagement PES frequency (e.g. “How often do you read news about science?,” 5-point Likert-type scale ranging from 0 = “never” to 5 = “almost daily,” Cronbach’s α = .71) and a multiple choice scale measuring 15 potential PES experiences during the last 12 months (e.g. “I know someone who does scientific research,” “I visited a science museum”). Finally, demographics (age, gender, occupation, academic discipline) and a “use-me” item (“Should we use your data for our analyses?,” yes/no) were assessed. Participants had the opportunity to participate in a lottery and sign up for more information and were debriefed.

#### Sample

Participants were recruited via mailing lists (e.g. by the university, by the research unit) and social networks (e.g. Facebook, science blogs). Inclusion criteria were very good German language skills and a minimum age of 16. A total of 521 participants completed the study. Applying pre-registered exclusion criteria (see https://osf.io/qja78), 184 participants had to be excluded from the dataset: 45 participants stated not to use their data; 34 participants spent less than 60 seconds viewing the manipulation; 105 participants failed the main manipulation comprehension checks.^4^ The final sample consisted of *N* = 337 participants (68.0% female, 32.0% male); ages ranged between 16 and 74 years (*M* = 43.33; *SD* = 14.73). Most participants were currently employed (59.6%; students: 20.2%; unemployed: 20.2%). Participants who were currently studying at a university or already had a university degree (61.4%) came from a variety of disciplines (law, economics, and social sciences: 33.2%; humanities: 10.7%; mathematics and natural sciences: 7.1%; engineering: 6.5%; medicine and life sciences: 3.3%). Although *N* = 337 is lower than the determined sample size specified in our pre-registration, the power is still large enough (i.e. 90%) to detect a small-to-medium interaction effect in our 2×3 analysis of variance (ANOVA; Φ^2^ = .195) on a 5% significance level (Faul et al., 2007).

### Results

Supporting the effectiveness of our randomization, neither general PES (PES frequency: *p* = .27; PES experiences: *p* = .61) nor prior knowledge about the replication debate (*p* = .12) differed between the six cells of our design (mean difference tests via one-way ANOVAs). Across all conditions, 19% of our participants had heard about the replication debate before; on average, they judged their knowledge about the replication debate (*M* = 4.03, *SD* = 1.52) and questionable research practices (QRPs; *M* = 3.34, *SD* = 1.61) to be moderate. Table 1 summarizes all means, standard deviations, and correlations.

**Table 1.** Means, standard deviations, and correlations between measured variables.

Variable		*M*	*SD*	Correlations								
				1	2	3	4	5	6	7	8	9
1	Expertise	4.79	0.95	.*94*								
2	Integrity/benevolence	4.47	1.00	.78**	.*95*							
3	Credibility	3.79	1.14	.73**	.75**	.*83*						
4	Willingness to engage	2.53	1.64	.29**	.27**	.36**	—					
5	Public funding support	4.00	1.33	.59**	.66**	.68**	.35**	—				
6	Likeability	3.69	1.31	.66**	.73**	.71**	.32**	.68**	—			
7	Replication debate knowledge	4.03	1.52	−.13	−.01	−.13	−.04	−.09	−.08	—		
8	QRPs knowledge	3.34	1.61	−.18	−.08	−.12	.02	−.16	−.16	.76**	—	
9	PES frequency	3.16	0.63	−.03	−.06	−.09	.16**	.01	−.07	−.09	−.07	.*71*
10	PES experiences	5.09	2.96	−.15**	−.15**	−.13	.09	–.05	–.07	.24	.25*	.51**

SD: standard deviation; QRP: questionable research practice; PES: public engagement with science.

*N* = 337; for variables 7 and 8: *N* = 64.

* *p* < .05; **p < .01. Cronbach’s α for each scale are reported in the diagonal (in italics).

Next, we conducted a 2×3 multivariate analysis of variance (MANOVA) to test the effects of self-criticism and reform intentions on the two epistemic trustworthiness dimensions (expertise and integrity/benevolence), credibility, and willingness to further engage with the research.^5^ Both self-criticism, *F*(4, 328) = 4.38, *p* < .01, Pillai-*V* = .05, η_p_^2^ = .05 (95% confidence interval (CI_95_) = .01; .09), and reform intentions, *F*(8, 658) = 20.53, *p* < .001, Pillai-*V* = .40, η_p_^2^ = .20 (CI_95_ = .14; .24), had multivariate main effects, while the interaction effect was not significant, *F*(8, 658) = .96, *p* = .46, Pillai-*V* = .02, η_p_^2^ = .01 (CI_95_ = .00; .02). We followed up with univariate analyses. Means and standard deviations, broken down by conditions, are reported in Table 2.

**Table 2.** Means and standard deviations, broken down by conditions.

Variable	Self-criticism	Reform intentions		
		None	Minor	Major
Expertise	No	4.00 (1.07)^a^	5.01 (0.84)^b^	5.14 (0.79)^b^
	Yes	4.30 (0.84)^a^	5.05 (0.72)^b^	5.22 (0.70)^b^
Integrity/benevolence	No	3.44 (0.94)^a^	4.65 (0.71)^b^	4.84 (0.83)^b^
	Yes	3.83 (0.85)^a^	4.95 (0.76)^b^	5.06 (0.67)^b^
Credibility	No	2.83 (1.15)^a^	4.26 (0.85)^b^	4.22 (0.87)^b^
	Yes	2.92 (0.96)^a^	4.18 (0.82)^b^	4.32 (0.94)^b^
Willingness to engage	No	2.12 (1.59)	2.91 (1.68)	2.67 (1.72)
	Yes	2.09 (1.53)^a^	2.35 (1.60)	3.05 (1.57)^b^

*N* = 337. Means (standard deviations in brackets). In each line, different letters in the superscript indicate significant pairwise differences (i.e. *p* < .05; Tukey honest significant difference (HSD) test).

#### Epistemic trustworthiness

Univariate analyses show a significant main effect of self-criticism on integrity/benevolence, *F*(1, 331) = 11.97, *p* < .001, η_p_^2^ = .03 (CI_95_ = .01; .08), but not on expertise, *F*(1, 331) = 2.39, *p* = .12, η_p_^2^ = .01 (CI_95_ = .00; .04), as well as significant main effects of reform intentions on both integrity/benevolence, *F*(2, 331) = 91.45, *p* < .001, η_p_^2^ = .36 (CI_95_ = .27; .42), and expertise, *F*(2, 331) = 50.62, *p* < .001, η_p_^2^ = .23 (CI_95_ = .16; .30). The interaction effects were non-significant on either dependent variable (DV; *p* = .44 for expertise; *p* = .71 for integrity/benevolence). Looking at the reform intentions factor, follow-up pairwise comparisons (i.e. Tukey honest significant difference (HSD) tests) suggest that both reform intention conditions differed from the no reform condition (all *p* < .001); yet, the extent of these reforms (i.e. minor vs major) did not affect the DVs (*p* = .35 for expertise; *p* = .37 for integrity/benevolence).

#### Credibility

On credibility, the main effect of self-criticism was not significant, *F*(1, 331) = .15, *p* = .70, η_p_^2^ < .001 (CI_95_ = .00; .02), while the main effect of reform intentions was significant, *F*(2, 331) = 78.72, *p* < .001, η_p_^2^ = .32 (CI_95_ = .24; .39). Again, there was no interaction effect, *F*(2, 331) = .28, *p* = .76, η_p_^2^ < .01 (CI_95_ = .00; .02). As before, the reform conditions significantly differed from the no reform condition (both *p* < .001); but the extent of reforms did not make a difference (*p* = .87).

#### Willingness to engage

On participants’ willingness to further engage with the research, we found no significant main effect of self-criticism, *F*(1, 331) = .14, *p* = .71, η_p_^2^ < .001 (CI_95_ = .00; .02), but a significant main effect of reform intentions, *F*(2, 331) = 6.85, *p* < .01, η_p_^2^ = .04 (CI_95_ = .01; .08). Once again, there was no significant interaction effect, *F*(2, 331) = 2.31, *p* = .10, η_p_^2^ = .01 (CI_95_ = .00; .04). Follow-up analyses reveal a slightly more complex pattern than before (see Table 2). Post hoc tests only revealed a significant difference between the no reform and the major reforms conditions on this DV (*p* < .001); all other comparisons were non-significant (no vs minor: *p* = .07; minor vs major: *p* = .35).

We also conducted explorative analyses to investigate the effects of self-criticism and reform intentions on public funding support as well as on likeability ratings. The results closely mirror the findings for credibility. These findings are not the focus of this article and are provided in a supplemental file (https://osf.io/yhsbp/). To scrutinize whether the effects reported here merely reflect an unspecific “halo” effect, we re-ran our analyses with likeability as a covariate (see supplemental file). Importantly, the main effect of self-criticism on integrity/benevolence remained significant, and the main effect of reform intentions on expertise, integrity/benevolence, and credibility remained significant, too. The main effect of reform intentions on willingness to engage, however, became non-significant. We will come back to this in the “General discussion” section.

### Discussion

These findings suggest that there are no detrimental effects of self-criticism and reform intentions on laypeople’s trustworthiness ascriptions and credibility perceptions. Expressing self-criticism (vs no self-criticism) led to higher benevolence and integrity perceptions—the affective dimensions of trustworthiness. Announcing reform intentions (compared to no reform intentions) had positive effects on epistemic trustworthiness and credibility, as well as on participants’ willingness to engage further with the expert’s research.

This pattern is in line with previous findings (Hendriks et al., 2016). Contradictorily, however, we found that self-criticism did not negatively affect expertise (Hendriks et al., 2016). This might be due to how self-criticism was particularly framed in our study: The self-critical researcher’s statement implied a more advanced methodological knowledge by the time he was interviewed compared to when the study was conducted (“according to my current methodological knowledge”), which actually suggests increased expertise that participants might have picked up upon. In Hendriks et al.’s (2016) study, however, the researcher revised his previous overgeneralizing statement in a blog entry, which might not have been perceived as improved knowledge but rather as correcting a careless mistake that could happen again.

Self-criticism only impacted affective dimensions of epistemic trustworthiness, but had no effect on expertise or on credibility. One reason for this could be that self-criticism regarding a particular study conducted in the past does not tell us much about the credibility of future research. A second reason could be that, in our study, reform intentions were mentioned at length at the end of the alleged interview, immediately before the DVs were measured. This might have overshadowed any effect of our self-criticism manipulation, which was mentioned earlier in the interview.

Announcing reforms had consistent and large effects on all of our DVs. Contrary to previous studies (Anvari and Lakens, 2018; Wingen et al., 2020), expressing even minor reform intentions led to higher ratings of epistemic trustworthiness and credibility compared to a no-reform intention control condition. Interestingly, the extent of such reforms did not have any effects. This suggests that it might be sufficient to signal at least some willingness to improve one’s scientific practices.

It should be noted that, in Study 1, self-criticism and reform intention statements were contrasted with conditions in which the researcher explicitly expressed no self-criticism regarding prior findings and/or refused implementing reforms. Thus, it is unclear whether our results display an increase in trust and credibility due to expressing self-criticism and/or reform intentions, a decrease of the same due to being overconfident, or both.

Our design allowed us to investigate whether self-criticism and reform intentions interact with each other. Regarding such an interaction, both amplifying effects (e.g. self-criticism makes reform intentions seem more reasonable) as well as alleviating effects (e.g. self-criticism alleviates the effect of reform intentions as they seem imperative) would have been plausible. However, we did not find any interaction effects on any of our measured variables, which suggests that self-criticism and reform intentions are independent of (and not contingent on) each other.

## 2. Study 2

Study 2 aimed to replicate and clarify the effects of self-criticism and reform intentions. For this purpose, we made some changes compared to Study 1. First, we manipulated each of the two independent variables, separately, because (a) we did not find evidence of an interaction between the two, and (b) doing so reduced the danger of any artificial “overshadowing” effects between the two manipulations. Second, to scrutinize which condition might drive the effects obtained in Study 1, we added neutral (“control”) conditions for both self-criticism and reform intentions (see “Methods” section). Third, we refined the operationalization of self-criticism as expression of a self-corrective attitude toward science: The researcher’s self-criticism was based on viewing his prior findings as preliminary and fragile (indicating high self-criticism) versus as fixed and definite (indicating lacking self-criticism). Fourth, we no longer differentiate between minor and major reform intentions, since Study 1 suggested no differences between these two conditions.

### Methods

#### Experimental manipulation

In Study 2, we again used a full between-subject design. However, we split the study in two parts (presented in randomized order): (2A) self-criticism (yes vs no vs no information) and (2B) reform intentions (yes vs no vs undecided). Participants read two alleged interviews with researchers, Dr Kugler and Dr Ecker, in which they talked about their own research on group processes (full materials are provided here: https://osf.io/yhsbp/).

In interview A, we manipulated self-criticism, defined as the expression of a self-critical attitude toward prior findings. When asked about a prior study, the researcher, Dr Kugler, described the results and then expressed either no self-criticism (“Still today, I actually do not have doubts about these findings. I see no reason why the results from back then shouldn’t also apply today and would view them as definite”), self-criticism (“However, today, I do have some doubts about these findings. Viewed scientifically, there might be reasons why the results from back then do not apply today and, thus, I would view them as preliminary”), or nothing of that kind (no information control condition).

In interview B, we manipulated reform intentions: After describing some general reform ideas in psychology and the ongoing debate, the interviewed researcher, Dr Ecker, either stated that he would not implement any reforms in his research (no reform intentions), that he would implement such reforms (reform intentions), or that he was still undecided whether to implement reforms (undecided).

#### Dependent variables

After each interview, we measured the same variables as in Study 1: First, they answered two respective attention checks (2A: “What was the optimal group size in Dr. Kugler’s earlier study?,” “Which topic would Dr. Kugler like to research next?;” 2B: “What is the goal of the discussed reforms according to Dr. Ecker?,” “Which topic would Dr. Ecker like to research next?”). Next, trustworthiness and credibility (as well as likeability and support for public funding) were measured exactly as in Study 1. In addition, we measured participants’ willingness to engage further with the researcher and his findings (one item used in Study 1 plus three additional items; for example, “I would like to learn more about Dr. Kugler’s/Dr. Ecker’s research;” assessed on a 6-point Likert-type scale ranging from 1 = “strongly disagree” to 6 = “strongly agree”).

#### Other measures

After each interview, we applied a comprehension check similar to Study 1 (2A: “Did Dr. Kugler express doubts about his prior findings?,” 2B: “What did Dr. Ecker say about his own intentions to implement reforms?”). In addition, after the interview concerning reforms in psychology (2B), we again asked whether participants had heard about the replication debate and, if yes, how extensive they judged their knowledge about the replication debate and QRP to be. Then, and similar to Study 1, we assessed participants’ general engagement with science (PES frequency and PES experiences; BBVA Foundation, 2011), demographics (age, gender, occupation, academic discipline), and a “use-me” item (“Do you think we should use your data for our analyses in this study?” yes/no). Finally, participants were fully debriefed and informed about their reimbursement.

#### Sample

As in Study 1, participants were recruited via mailing lists and social networks and could participate when they were older than 16 years, had very good German language skills, and had not previously participated in Study 1. We collected data from 400 participants as prescribed by our preregistered a priori power analyses for each study part based on our findings in Study 1 (see https://osf.io/9szde/). Applying our preregistered exclusion criteria, 35 participants had to be generally excluded:^6^ 11 participants denied the “use-me” question; 5 participants fell below the minimum threshold of 30 seconds for viewing the manipulation texts; 4 participants completed the questionnaire in less than 5 minutes; 15 participants failed the comprehension check in both parts. The final sample consisted of *N* = 365 participants; ages ranged between 16 and 77 years (*M* = 30.60; *SD* = 13.95; 81.10% female, 18.63% male, 0.27% other). A majority was currently enrolled in a higher education program (64.1%; employed: 26.8%; unemployed: 9.0%). Participants who were studying at a university or already had a university degree (90.1%) came from a variety of disciplines (law, economics, and social sciences: 58.2%; humanities: 9.2%; mathematics and natural sciences: 9.7%; engineering: 3.3%; medicine and life sciences: 6.7%). Thus, compared to Study 1, the sample in this study was younger, more female, and more highly educated.

### Results

Supporting the effectiveness of our randomization, neither general PES (PES frequency: *p_a_* = .58 and *p_b_* = .63; PES experiences: *p_a_* = .94 and *p_b_* = .70) nor prior knowledge about the replication debate (*p_a_* = .81 and *p_b_* = .76; overall, 34% had heard of it before) differed between experimental conditions in any of the two study parts (mean differences tested via one-way ANOVAs). Descriptive statistics and correlations between measured variables are reported in Tables 3 (for Study 2A) and 4 (for Study 2B).

**Table 3.** Means, standard deviations, and correlations between measured variables for study 2A (self-criticism).

Variable		*M*	*SD*	Correlations								
				1	2	3	4	5	6	7	8	9
1	Expertise	4.93	0.87	.*93*								
2	Integrity/benevolence	4.68	0.88	.73**	.*95*							
3	Credibility	4.05	0.98	.64**	.63**	.*77*						
4	Willingness to engage	3.74	1.14	.51**	.48**	.47**	.*77*					
5	Public funding support	4.44	1.11	.59**	.61**	.56**	.51**	—				
6	Likeability	4.24	1.24	.66**	.77**	.58**	.54**	.64**	—			
7	Replication debate knowledge	3.61	1.50	.11	.12	.07	.04	.03	.07	—		
8	QRPs knowledge	3.06	1.66	.04	.08	.07	−.01	.04	.01	.83**	—	
9	PES frequency	3.10	0.68	−.01	−.03	−.04	.05	.07	−.06	.15	.17	.*73*
10	PES experiences	6.15	2.75	−.06	−.09	−.10	.01	.03	−.10	.12	.09	.45**

SD: standard deviation; QRP: questionable research practice; PES: public engagement with science.

*N* = 288; for variables 7 and 8: *N* = 193.

**p < .01. Cronbach’s α for each scale are reported in the diagonal (in italics).

**Table 4.** Means, standard deviations, and correlations between measured variables for study 2B.

Variable		*M*	*SD*	Correlations								
				1	2	3	4	5	6	7	8	9
1	Expertise	4.98	0.82	.*93*								
2	Integrity/benevolence	4.47	0.96	.71**	.*95*							
3	Credibility	3.85	0.97	.54**	.69**	.*76*						
4	Willingness to engage	3.51	1.18	.31**	.37**	.41**	.*81*					
5	Public funding support	4.23	1.21	.58**	.66**	.65**	.40**	—				
6	Likeability	3.94	1.23	.56**	.67**	.60**	.52**	.69**	—			
7	Replication debate knowledge	3.69	1.45	−.10	−.23*	−.23*	−.12	−.20*	−.20*	—		
8	QRPs knowledge	3.07	1.66	−.16	−.26**	−.27**	−.09	−.21*	−.21*	.80**	—	
9	PES frequency	3.15	0.67	.00	.02	−.07	.05	−.02	−.06	.14	.11	.*73*
10	PES experiences	6.37	2.75	.01	−.02	−.09	.06	.02	−.06	.15	.10	.46**

SD: standard deviation; QRP: questionable research practice; PES: public engagement with science.

*N* = 322; for variables 7 and 8: *N* = 203. *p < .05; **p < .01. Cronbach’s α for each scale are reported in the diagonal (in italics).

#### Self-criticism (Study 2A)

Using a MANOVA, we tested the effects of self-criticism on the two epistemic trustworthiness dimensions (expertise and integrity/benevolence), credibility, and willingness to engage: The multivariate main effect of self-criticism was, again, significant, and slightly larger than in Study 1, *F*(8, 566) = 6.38, *p* < .001, Pillai-*V* = .17, η_p_^2^ = .08 (CI_95_ = .03; .12). We followed up with univariate analyses. Self-criticism had a significant effect on all our DVs: integrity/benevolence, *F*(2, 285) = 24.96, *p* < .001, η_p_^2^ = .15 (CI_95_ = .08; .22), expertise, *F*(2, 285) = 15.24, *p* < .001, η_p_^2^ = .10 (CI_95_ = .04; .16), credibility *F*(2, 285) = 14.76, *p* < .001, η_p_^2^ = .09 (CI_95_ = .04; .16), and willingness to engage, *F*(2, 285) = 6.04, *p* < .01, η_p_^2^ = .04 (CI_95_ = .01; .09). Follow-up pairwise comparisons (i.e. Tukey HSD tests) show that expressing no self-criticism compared to self-criticism or no information led to significantly lower mean values on all DVs. There were no significant differences, however, between expressing self-criticism versus giving no information (i.e. the control condition). Means and standard deviations, broken down by conditions, and results for follow-up tests are reported in Table 5 (upper part).

**Table 5.** Means and standard deviations, broken down by conditions for study A (self-criticism) and Study B (reform intentions).

Variable	Self-criticism (Study 2A)		
	No self-criticism	No information	Self-criticism
Expertise	4.56 (0.96)^a^	5.10 (0.72)^b^	5.14 (0.77)^b^
Integrity/benevolence	4.23 (0.98)^a^	4.79 (0.75)^b^	5.00 (0.69)^b^
Credibility	3.63 (1.05)^a^	4.24 (0.78)^b^	4.28 (0.93)^b^
Willingness to engage	3.42 (1.09)^a^	3.90 (1.17)^b^	3.92 (1.11)^b^
	Reform intentions (Study 2B)		
	No reform intentions	Undecided	Reform intentions
Expertise	4.83 (0.94)^a^	4.86 (0.77)^a^	5.22 (0.68)^b^
Integrity/benevolence	4.03 (1.04)^a^	4.38 (0.84)^b^	4.98 (0.71)^c^
Credibility	3.46 (1.04)^a^	3.76 (0.93)^b^	4.31 (0.73)^c^
Willingness to engage	3.24 (1.23)^a^	3.61 (1.13)	3.70 (1.14)^b^

Study 2A: *N* = 288; Study 2B: *N* = 322. Means (standard deviations in brackets). In each line, different letters in the superscript indicate significant pairwise differences (i.e. *p* < .05; Tukey honest significant difference (HSD) test).

#### Reform intentions (Study 2B)

Again, using a MANOVA, we tested the effects of reform intentions on epistemic trustworthiness (expertise and integrity/benevolence), credibility, and willingness to engage. The multivariate main effect of reform intentions was, again, significant, yet slightly smaller than in Study 1, *F*(8, 634) = 9.87, *p* < .001, Pillai-*V* = .22, η_p_^2^ = .11 (CI_95_ = .06; .15). We followed up with univariate analyses. Reform intentions had a significant effect on all our DVs: integrity/benevolence, *F*(2, 319) = 33.42, *p* < .001, η_p_^2^ = .17 (CI_95_ = .10; .24), expertise, *F*(2, 319) = 7.90, *p* < .001, η_p_^2^ = .05 (CI_95_ = .01; .10), credibility, *F*(2, 319) = 25.05, *p* < .001, η_p_^2^ = .14 (CI_95_ = .07; .20), and willingness to engage, *F*(2, 319) = 4.85, *p* < .01, η_p_^2^ = .03 (CI_95_ = .00; .07). Follow-up tests showed that for all DVs, reform intentions compared to no reform intentions led to significantly higher mean values. Reform intentions compared to being undecided led to significantly higher mean values on all variables except for willingness to engage. And being undecided compared to no reform intentions led to significantly higher mean values on all variables except for expertise and, again, willingness to engage. Means and standard deviations, broken down by conditions, and results for follow-up tests are reported in Table 5 (lower part).

Again, explorative analyses of the effects of self-criticism and reform intentions on public funding support as well as likeability indicate a very similar pattern of results as described above for our DVs (see https://osf.io/yhsbp/). When controlling for likeability in our analyses to scrutinize a possible halo-effect, self-criticism only had a significant effect on integrity/benevolence, and reform intentions only had significant effects on integrity/benevolence and credibility. We will come back to this in the “General discussion” section.

### Discussion

Study 2 replicates and further qualifies the results from Study 1. Again, our findings indicate no harm in being self-corrective. Actually, Study 2 suggests that there is harm in not being self-corrective: While researchers’ expression of a self-critical attitude toward their previous findings (compared to no such expression) did not affect trustworthiness (expertise and benevolence/integrity), perceived credibility, or willingness to further engage with this research, researchers who expressed no self-criticism and presented their findings as fixed and definite were perceived as less trustworthy and less credible, and participants were less willing to engage with this research (compared to a neutral control condition). Whereas in Study 1, the researcher in the self-criticism condition expressed doubts about his prior finding as a “second thought” regarding his methodology, Study 2 operationalized self-criticism as being mindful of the fragility and preliminary nature of (his) research, in general. Researchers who deny this fragility and tentativeness of science might not only be perceived as less benevolent and integer, but also as less competent (as this attitude contradicts the basic idea of science as being self-corrective), which, in turn, questions the quality of their future research and makes them seem less of exemplary researchers.

Regarding reform intentions, our findings indicate a benefit of being self-corrective: Announcing reform intentions (compared to being undecided and/or intending no reforms) increased perceived trustworthiness (expertise and benevolence/integrity) and credibility of their future research, and led participants to report a higher willingness to further engage with this research. Nevertheless, we also find that dismissing reforms can harm the public’s trust in science: When the researcher announced not to implement reforms, integrity/benevolence and credibility ratings were considerably lower. Although the effects of reform intentions in Study 2 were smaller than in Study 1, our findings support the same conclusion: Researchers’ expressed positions on reform intentions have the potential of enhancing (as well as impairing) the public’s trust and interest to engage with science.

## 3. General discussion

In two studies, we demonstrate the effects of two ways of being self-corrective in science: expressing self-criticism and intending to implement reforms. Our findings suggest that there is no harm in expressing criticism toward one’s own research or in announcing one will implement reforms. In fact, such self-corrective behavior was superior to non-corrective behavior in terms of laypeople’s perceptions of researchers’ epistemic trustworthiness (expertise and integrity/benevolence) and the perceived credibility of their research, and led to a higher willingness to further engage with researchers and their findings.

Researchers’ self-criticism (i.e. reflecting critically upon prior studies and regarding scientific findings as preliminary and fragile) did not have negative effects. However, explicitly expressing a lack of doubts impaired trustworthiness (especially integrity and benevolence), credibility, and even participants’ willingness to further engage with such research. Thus, in the eyes of the public, self-criticism does not harm, while a lack of self-criticism does. This has important implications for researchers communicating their findings: Openly expressing uncertainties and acknowledging the inherent preliminary nature of new scientific findings seems unproblematic for researchers’ reputation. However, on the contrary, appearing overconfident might have considerable reputational costs.

Across both studies, researchers’ reform intentions (i.e. planning to implement currently discussed reforms in future research) consistently led to more trust in and willingness to engage with science. Interestingly, these effects were driven both by the intention to implement reforms and, in the reverse direction, by an explicit dismissal of such reforms. In addition, Study 1 suggests that the extent to which researchers are willing to implement reforms (i.e. minor vs major reforms) does not play a decisive role for their public perception. These findings cast a new light on psychological research on the public perception of reforms in psychology: contrary to previous studies (Anvari and Lakens, 2018; Wingen et al., 2020), participants reacted quite positively to the idea of implementing reforms in science. Our studies extend this prior research in two important ways: First, previous studies focused on transparency as the main aspect of science reforms, but did not explain in detail how this connects to more reliable and credible research. One could argue that the connection is obvious; yet, explaining the link between transparency and higher reliability in more detail (and simpler words) to participants may have contributed to the positive effects of reform intentions on trustworthiness and credibility that we found in our studies. This also has important implications for communicating science reforms to the public: focusing on the superordinate goal these reforms aim to achieve (instead of merely portraying these reforms to be good for their own sake) might help lay audiences understand what they are about and why they are relevant. That said, it should be noted that we did not really measure participants’ understanding of the consequences of science reforms for science as a whole; instead, we focused on participants’ epistemic trustworthiness in one particular scientist. Thus, future research should look at how such individualized trustworthiness perceptions may generalize onto trust in science and the perceived credibility of science as a whole.

As scientific progress is not a solitary endeavor but a collaborative effort, researchers might also worry about their colleagues’ perceptions of them. Future research should investigate how other researchers perceive their self-corrective peers. In fact, first evidence suggests that researchers receive wrongness admission of their colleagues positively (Fetterman and Sassenberg, 2015) and that, following (self-)correction, they indeed update their scientific beliefs in light of such new evidence (yet, not as much as they should; McDiarmid et al., 2021).

Our findings suggest that researchers are perceived as more trustworthy and their research as more credible when they express self-criticism and reform intentions. One might argue that this pattern reflects nothing more than a positive acknowledgment of other people’s humility (Chancellor and Lyubomirsky, 2013; Powers and Zuroff, 1988). However, it should be noted that many of our findings persisted after controlling for general likeability of the target person (i.e. the researcher in Studies 1 and 2), even though likeability ratings were highly correlated with trustworthiness (see Tables 1, 3, and 4): integrity/benevolence ascriptions at least can, thus, not be explained by such a “halo” effect. However, likeability strongly predicted participants’ willingness to engage with the research and also suppressed the significant main effects of self-criticism and reform intentions on this DV in both studies. This suggests that the extent to which laypeople are motivated to learn more about science is contingent on their overall impression of a scientist. Importantly, however, laypeople’s ascriptions of integrity and benevolence—the “affective” dimensions of epistemic trustworthiness (Hendriks et al., 2015; McAllister, 1995)—are specifically affected by expressions of self-criticism and reform intentions, irrespective of more general likeability ratings.

## 4. Conclusion

Our findings suggest that researchers’ hesitation toward self-correction (e.g. Fetterman and Sassenberg, 2015; Frewer et al., 2003; Rohrer et al., 2021; van der Bles et al., 2020) seems unwarranted: there is no harm in openly admitting doubts and regarding one’s findings as preliminary or in intending to reform one’s work routines. On the contrary, researchers who portray their findings as fixed and definite and who are unwilling to implement reforms are perceived as less trustworthy and less credible by laypeople. In this regard, the current discussion of self-criticism and reforms (e.g. the open science movement) might prove to be an attention-drawing door opener for greater lay engagement with science; a chance for science to improve not only its methodological rigor, but also its relationship with the public.

## Notes

1. Willingness to engage was explored in Study 1 and preregistered only in Study 2.

2. We made “Dr. Romberg” (Study 1), as well as “Dr. Kugler” and “Dr. Ecker” (Study 2) male researchers because of linguistic simplicity in the German language, yet, we assume the manipulations would work just as well with female researchers. We avoided overemphasizing gender by using the neutral “Dr. Romberg/Kugler/Ecker” more that “he/his/him.”

3. Contrary to the proposed three-factor solution by Hendriks et al. (2015), factor analyses conducted with the present data suggest a two-factor solution with expertise (factor 1) and integrity/benevolence (factor 2), corresponding to the idea of a cognitive and an affective dimension of trustworthiness. Deviating from our pre-registration in Study 1, epistemic trustworthiness will be analyzed on these two dimensions instead of three.

4. Furthermore, 83 participants could not correctly remember the extent of the reform intention but, in line with the preregistration, were not excluded. Excluding them did not change the results on our dependent variables (DVs). Deviating from the preregistration, we did not exclude 56 participants with same answers on every single item on a questionnaire page or choosing only extreme response options (regarding epistemic trustworthiness and credibility). Excluding them did not change our results on our DVs. Furthermore, one could argue that these are, in fact, plausible answers. Because of this and additional power concerns, we decided not to exclude these participants.

5. Because of the very high intercorrelations, we deviated from the preregistered analyses by adding all DVs to the MANOVA. The results did not differ significantly from the originally planned analyses.

6. We describe the sample with a general dataset including participants who fulfilled the criteria for at least one part of the study. For specific analyses in each part of the study, we only used data from participants who fulfilled the criteria for the respective part of the study (*N*_2A_ = 288; *N*_2B_ = 322).
